# MRS2 and mitochondrial gene networks in endometrial cancer: mechanisms, biomarkers, and therapeutic implications

**DOI:** 10.7717/peerj.20409

**Published:** 2026-01-12

**Authors:** Leyi Huang, Yafang Kang, Wenyu Lin, Jiaxi Chen, Yuxuan Huang, Yan Zhang, Yihan Shen, Zeyu Wu, Sihao Chen, Shaoqing Zheng, Yiyang Wang, Renxi Lin, Yuanlin Qi

**Affiliations:** 1 Laboratory of Gynecologic Oncology, Fujian Maternity and Child Health Hospital, College of Clinical Medicine for Obstetrics & Gynecology and Pediatrics, Fujian Medical University, Fuzhou, Fujian, China; 2 Department of Biochemistry and Molecular Biology, Fujian Medical University, Fuzhou, Fujian, China; 3 Medical Genetic Diagnosis and Therapy Center, Fujian Maternity and Child Health Hospital, College of Clinical Medicine for Obstetrics & Gynecology and Pediatrics, Fujian Medical University, Fujian Key Laboratory for Prenatal Diagnosis and Birth Defect, Fuzhou, Fujian, China; 4 The State Key Laboratory of Molecular Vaccinology and Molecular Diagnostics, National Institute of Diagnostics and Vaccine Development in Infectious Diseases, School of Public Health, Xiamen University, Xiamen, Fujian, China; 5 Experimental Teaching Center of Basic Medicine, Fujian Medical University, Fuzhou, China

**Keywords:** MRS2, Magnesium ion, Mitochondrion, ROS, Endometrial cancer

## Abstract

Magnesium ions and their transport proteins are increasingly recognized for their critical roles in tumor progression. However, their specific mechanisms in endometrial cancer (EC) remain poorly understood. This study investigated the role of Mitochondrial RNA Splicing 2 protein (MRS2), a key mitochondrial magnesium transporter, and its associated genes, in regulating mitochondrial function and the invasive and metastatic capabilities of EC cells. Using a combination of experimental approaches including lactate detection, flow cytometry, immunofluorescence, CCK8 assays, and Transwell migration assays, along with bioinformatics analysis, we investigated the relationship between lactate levels and MRS2 expression in endometrial cancer cells (KLE). Our findings suggest that elevated lactate levels are associated with increased MRS2 expression in mitochondria. This correlation was further linked to enhanced reactive oxygen species (ROS) production and altered expression of mitochondrial-related genes. Notably, MRS2 knockdown resulted in reduced proliferation of KLE cells, supporting a potential functional role of MRS2 in endometrial cancer progression. These findings provide new insights into the molecular mechanisms underlying EC progression and highlight MRS2 as a potential therapeutic target.

## Introduction

Endometrial carcinoma (EC) remains a significant global health concern, ranking as the sixth most common malignant tumor in women and the most prevalent gynecologic malignancy, accounting for approximately 7% of all female malignancies. In 2020, there were an estimated 417,000 new cases and 97,000 deaths attributable to EC (Siegel et al., 2022; Sung et al., 2021). Despite advances in early diagnosis and treatment, the prognosis for advanced or recurrent EC remains poor, with a 5-year survival rate below 20% (Brooks et al., 2019). Current treatment modalities, including surgery, chemotherapy, radiation therapy, and brachytherapy, often fail to prevent the progression to more aggressive forms of the disease (Lu & Broaddus, 2020). The current prognostic evaluation system based on clinical, pathological, imaging, and biological features exhibits limited ability to accurately predict the development and prognosis of EC (Lee et al., 2021).

According to the well-known Warburg effect, tumor cells preferentially utilize glycolysis for energy production, which results in an accumulation of lactate in the tumor microenvironment. Furthermore, studies have revealed that lactate plays multiple roles in cancer cell metabolism. It is not only a byproduct of glycolysis but also serves as a critical energy source for cancer cells (Sun et al., 2025). Its accumulation in the tumor microenvironment influences tumor cell growth and metastasis (Chen et al., 2024). Additionally, lipopolysaccharide (LPS) stimulation can shift cellular metabolism toward glycolytic dependency, a metabolic alteration that directly enhances lactate production (Caslin et al., 2019). The elevated lactate levels may further promote cancer proliferation by supplying energy and remodeling the tumor microenvironment.

Elevated lactate in the tumor microenvironment disrupts mitochondrial function, promotes histone lactylation, and enhances reactive oxygen species (ROS) production, which collectively contribute to the upregulation of oncogenic proteins such as L1CAM and ultimately accelerate tumor progression (Giannini et al., 2024; Vizza et al., 2021). The broader role of mitochondrial dysfunction, particularly in metabolic reprogramming and ROS regulation, in cancer progression, has attracted broad interest. However, the specific function of the Mitochondrial RNA Splicing 2 protein (MRS2), a key mitochondrial magnesium channel, in EC pathogenesis and its potential as a therapeutic target remain largely unexplored (Su et al., 2022b).

Cellular adenosine triphosphate (ATP) is mainly produced by mitochondrial metabolism, and the metabolic reprogramming of tumor cells requires the involvement of mitochondria (Guo, 2021). Beyond their role in energy production, mitochondria serve as a reservoir for apoptotic factors, linking them to a key hallmark of cancer: the evasion of cell death. It has been widely reported that both apoptosis and ferroptosis are mediated by mitochondria through various biological processes such as the generation of ROS, lipid peroxidation, and the intrinsic mitochondrial apoptotic signaling pathway (Battaglia et al., 2020; Yu et al., 2017). Therefore, mitochondria, as crucial organelles for cellular metabolism and cell death, represent a potential target for the development of anticancer therapies (Battaglia et al., 2020; Ghosh et al., 2020).

MRS2 and the solute carrier family 41 member 3 (SLC41A3) have been established as the only two magnesium transport proteins located on the inner mitochondrial membrane (IMM) in human cellular mitochondria. MRS2 belongs to the large, heterogeneous corA/MRS2/Alr1 super-family, characterized by highly conserved GMN motifs at the C-terminal end of its first transmembrane helix. This motif is crucial for protein function, where mutations either completely abolish magnesium ions (Mg^2+^) transport or alter the ion selectivity of the channel (Khan et al., 2013; Kolisek et al., 2003; Kubota et al., 2005; Mastrototaro et al., 2016).

As the second most abundant cation in the human body, magnesium ions play a crucial role in life processes, including cellular growth, differentiation, energy metabolism, and neuronal and cardiac excitability through the regulation of enzyme-catalyzed reactions (De Baaij, Hoenderop & Bindels, 2015). Acting as a calcium ion antagonist, magnesium ions can regulate cellular mitochondrial energy metabolism by modulating the flow between subcellular organelles, thereby further influencing cell survival (McStay, 2017; Daw et al., 2020). Magnesium ions and their transporters significantly influence tumor development. As a key cofactor for mitochondrial metabolic enzymes, magnesium deficiency caused by transporter inhibition impairs electron transport chain activity, inducing oxidative stress and promoting tumor cell apoptosis (Huang et al., 2024). *In vivo*, MagT1 knockout suppresses breast cancer progression by reducing Ki67 expression (Li et al., 2022). Consistently, clinical studies associate higher magnesium intake with improved survival in colorectal cancer (Wesselink et al., 2020). This suggests that magnesium-regulated mitochondrial metabolism could serve as a key factor in the diagnosis and treatment of energy metabolism-related cancers.

As an important transporter that regulates the spatiotemporal redistribution of intracellular magnesium ions (from the endoplasmic reticulum to mitochondria), MRS2 plays a crucial role in cellular metabolism (Daw et al., 2020). Tumor cells exhibit a preference for glycolysis to metabolize glucose and produce substantial lactate, even under oxygen-sufficient conditions, a phenomenon known as the Warburg effect. Since glycolysis is less efficient in ATP production, tumor cells undergo adaptive changes in mitochondrial function and activity to compensate for their high energy demands (Shirmanova, Sinyushkina & Komarova, 2023). Given that MRS2 regulates mitochondrial function in lactate-rich microenvironments, we hypothesize that it may play a critical regulatory role in energy metabolism-associated cancers such as endometrial cancer. However, the precise underlying mechanism remains elusive (Daw et al., 2020).

As a significant hormone-related tumor, the incidence and progression of endometrial cancer are closely associated with mitochondrial-regulated energy metabolism (Onstad, Schmandt & Lu, 2016). Metabolic disorders such as obesity and dyslipidemia have been established to be independent risk factors for endometrial cancer (Onstad, Schmandt & Lu, 2016). However, the precise mechanisms underlying these associations remain incompletely understood. MRS2 and its related genes may serve as key regulatory genes for mitochondrial metabolic abnormalities in endometrial cancer. Overall, the present study sought to explore the regulatory mechanisms of MRS2 and mitochondria-related genes in the progression of endometrial cancer, providing new insights for guiding early screening of EC patients, rational immunotherapy, and the development of clinical drugs for this patient population.

## Results

### Lactic acid increases ROS production by activating MRS2

It is well-established that tumor cells frequently exhibit a preference for aerobic glycolysis due to the Warburg effect, leading to significant lactate accumulation within the tumor microenvironment. Furthermore, emerging research indicates that lactate enrichment stimulates tumor proliferation, invasion, and immune evasion (Zong et al., 2024). Consequently, monitoring lactate content serves as an effective indicator for assessing tumor proliferation levels.

**Figure 1:**
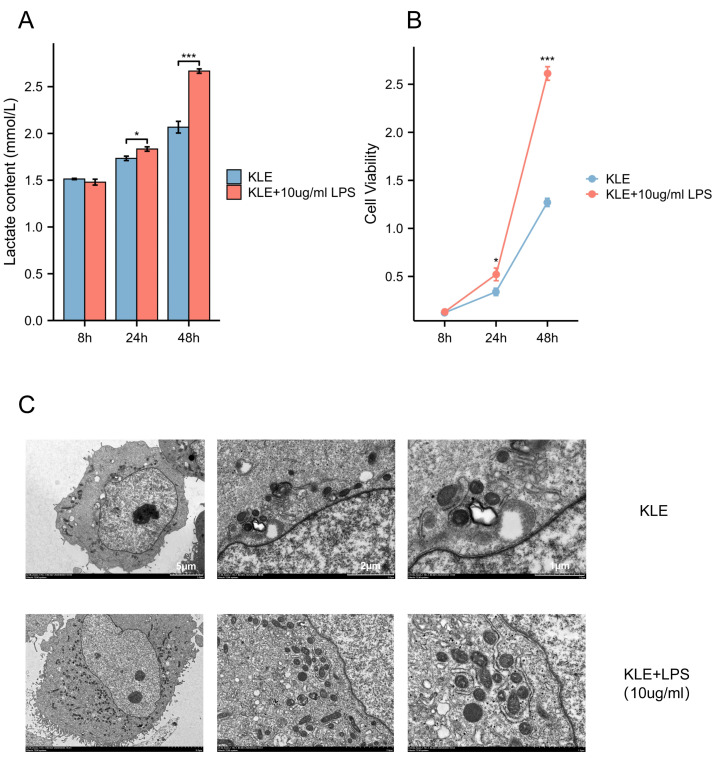
Lactic acid increases ROS production by activating MRS2. (A) Lactate content in control group (KLE) and LPS group (KLE+10 ug/ml LPS). (B) KLE cell was treated with LPS (10 ug/ml) for 48 h; then, cell viability was assessed using the CCK8 assay. (C) Representative transmission electron microscopy (TEM) images of control and LPS-treated KLE cells. The upper and lower panels show ultrastructural features of untreated KLE cells and cells treated with 10 µg/mL LPS for 24 h, respectively. LPS-induced alterations in mitochondrial morphology and quantity are evident in the treated group. Scale bars: five µm, two µm, and one µm (as indicated on the corresponding panels). (^∗^ indicated *p* ≤ 0.05, ^∗∗∗^ indicated *p* ≤ 0.001).

**Figure 2:**
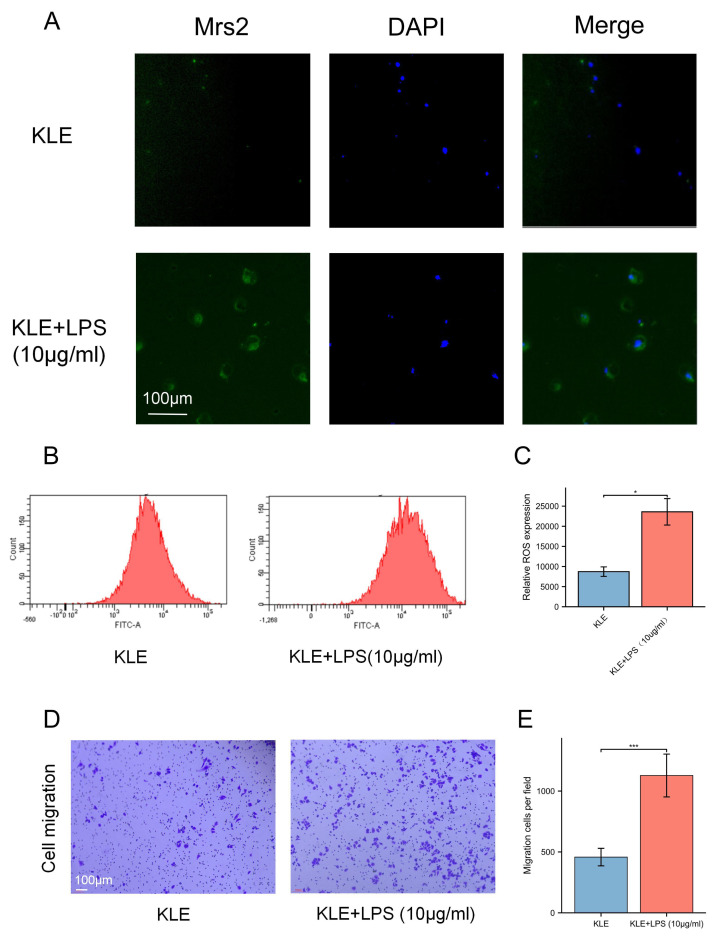
Lactic acid increases ROS production by activating MRS2. (A) The expression of MRS2 was imaged using florescence microscopy after LPS treatment for 48 h, scale bar: 100 µm. (B, C) Intracellular ROS production was measured by flow cytometry using DCFH-DA staining after treating KLE cell with LPS (10 ug/ml) for 48 h. The histogram compares ROS levels between KLE cells and KLE cells with LPS treatment. (D, E) The migration abilities of EC cells after LPS (10 ug/ml) treatment for 48 h, and statistical analysis results (magnification, 200×; scale bars, 100 µm). (^∗^ indicated *p* ≤ 0.05, ^∗∗∗^ indicated *p* ≤ 0.001).

To explore the potential effects of lactate levels on cancer cell metabolism in response to LPS stimulation and to validate the functional role of MRS2 in endometrial cancer, we performed a series of *in vitro* experiments, including lactate detection, ROS measurement, cell proliferation assays, and transwell migration assay. Our findings revealed that increased lactate levels in endometrial cancer cells (KLE) after LPS stimulation, along with upregulated MRS2 expression (Fig. 1A). Immunofluorescence staining showed that MRS2 (green fluorescence) exhibited a weak signal in KLE cells, which was significantly enhanced in the KLE + LPS group cells. The merged image, a superposition of MRS2 and nuclear staining, clearly demonstrated that LPS stimulation could promote the expression of MRS2 in KLE cells (Fig. 2A). Flow cytometry analysis revealed that LPS-stimulated KLE cells (10 µg/ml, 48 h) produced higher levels of ROS (Figs. 2B–2C). Moreover, CCK assays (Fig. 1B) and Transwell migration tests (Figs. 2D–2E) confirmed that these cells exhibited stronger proliferative and invasive tendencies (*p* < 0.05). Finally, transmission electron microscopy (TEM) analysis revealed that KLE cells treated with LPS for 24 h exhibited a significant increase in mitochondrial number compared to the control group (Fig. 1C). Collectively, our observations indicate a potential association between lactate enrichment and MRS2 upregulation in KLE cells. This MRS2 induction appears to impact mitochondrial function, correlating with increased ROS levels and enhanced tumor proliferative/invasive activity. These findings support a possible mechanistic link whereby lactate may promote tumor progression through MRS2-mediated mitochondrial dysfunction and subsequent ROS generation, ultimately influencing cancer cell proliferation and invasion.

### MRS2 drives endometrial cancer malignancy

**Figure 3:**
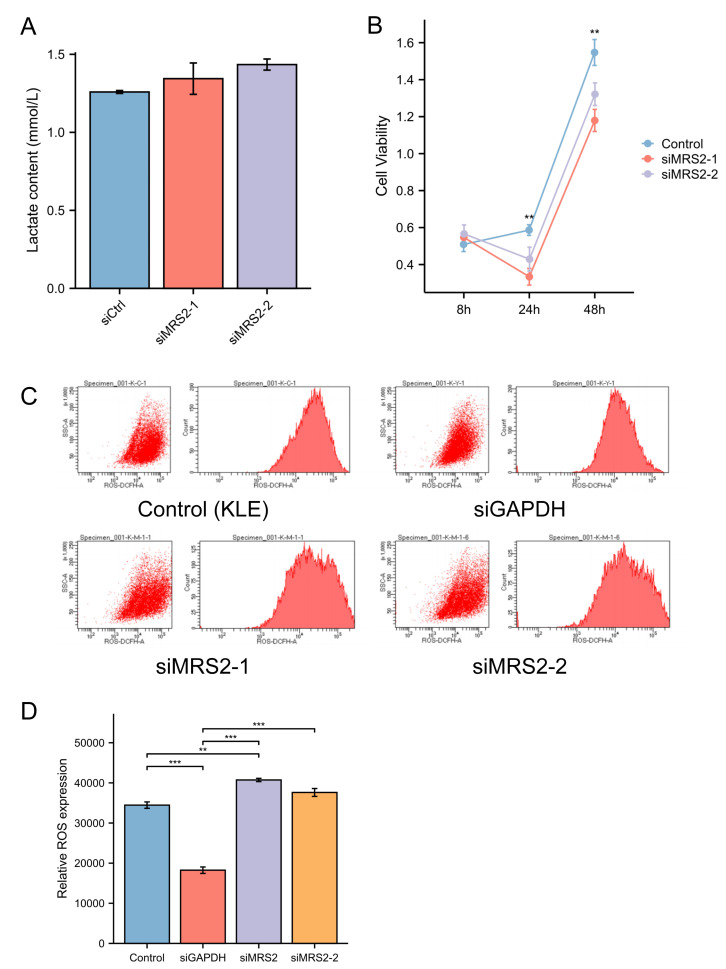
MRS2 drives endometrial cancer malignancy. (A) Lactate content in control group (KLE) and LPS group (KLE+10 ug/ml LPS) after 24 h. (B) Assessment of cell proliferation in KLE cells following MRS2 knockdown. Cell viability was measured using CCK-8 assay after 48 h of culture. The results compare proliferation rates between control KLE cells and cells transfected with MRS2-targeting siRNA (siMRS2). (C, D) Detection of ROS levels in KLE cells following MRS2 knockdown. Intracellular ROS production was measured by flow cytometry using DCFH-DA staining after 48 h of culture. The histogram compares ROS levels between control KLE cells and cells transfected with MRS2-targeting siRNA (siMRS2). (^∗∗^ indicated *p* ≤ 0.01, ^∗∗∗^ indicated *p* ≤ 0.001).

To further investigate the functional role of MRS2 in endometrial cancer progression, we generated an MRS2-knockdown KLE cell model using siRNA. Interestingly, MRS2 knockdown did not significantly alter lactate levels in the cell supernatant, suggesting that LPS—but not MRS2—is the primary inducer of lactate production in endometrial cancer cells, as supported by earlier findings in ‘Lactic acid increases ROS production by activating MRS2’ (Fig. 3A).

Subsequent flow cytometry analysis revealed a notable increase in ROS levels in MRS2-knockdown cells compared to controls (Fig. 3B). Furthermore, CCK-8 assays demonstrated that cell viability was significantly reduced in the siRNA-MRS2 group at both 24 and 48 h relative to the control group (*p* < 0.05) (Fig. 3C). These results collectively indicate that MRS2 expression is positively associated with proliferative capacity in endometrial cancer cells.

### Elevated MRS2 expression in endometrial cancer correlates with poor prognosis

**Figure 4:**
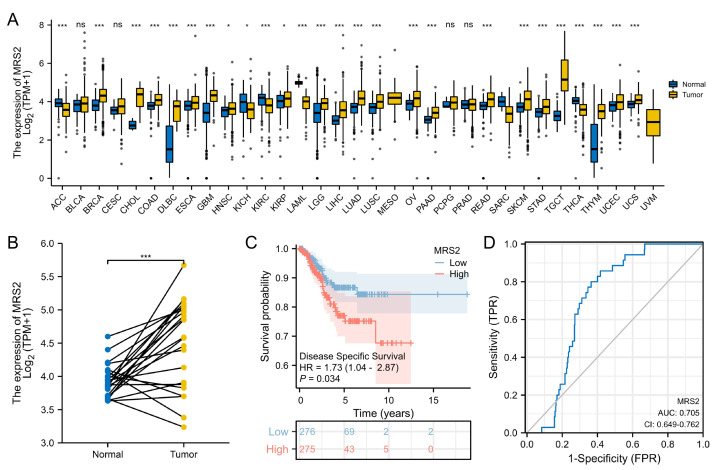
Elevated MRS2 expression in endometrial cancer correlates with poor prognosis. (A) Expression differences of MRS2 in various tumor tissues and normal tissues. (B) Expression differences of MRS2 in endometrial cancer tissues and normal tissues. (C) Kaplan–Meier curve for disease specific survival. High and low expression were categorized according to the median expression value as a threshold, with patients in the high-expression group having MRS2 levels above the median and those in the low-expression group having MRS2 levels below the median. (D) Receiver operating characteristic (ROC) curve for MRS2. The ROC curve demonstrates the sensitivity and specificity of MRS2 expression levels for distinguishing endometrial cancer tissues from normal controls. The area under the curve (AUC) value of 0.705 indicates moderate diagnostic accuracy. (ns indicated *p* > 0.05, ^∗^ indicated *p* ≤ 0.05, ^∗∗^ indicated *p* ≤ 0.01, ^∗∗∗^ indicated *p* ≤ 0.001, ^∗∗∗∗^ indicated *p* ≤ 0.0001).

The RNA-seq data were downloaded from UCSC XENA (https://xenabrowser.net/datapages/) and subjected to uniform analysis. The normality test was performed on the FPKM data of the training set, yielding a *p*-value of 0.06726. As this value was greater than 0.05, the data were considered to be normally distributed. Analysis of TCGA data revealed significant upregulation of MRS2 in 21 tumor types, including endometrial cancer. High MRS2 expression correlated with poor tumor differentiation and worse prognosis (Table 1). MRS2 was upregulated across tumor tissues (Fig. 4A); in endometrial cancer, there was a significant difference in its expression between cancerous tissues and normal tissues (Fig. 4B). K-M curve analysis showed that high MRS2 expression was associated with low survival (Fig. 4C). ROC curve analysis yielded an area under the curve (AUC) of 0.705 for MRS2 (Fig. 4D), demonstrating favorable diagnostic efficacy and suggesting its potential as a diagnostic biomarker for endometrial cancer (Fig. 4D).

**Table 1:** Clinical characteristics of EC patients from TCGA database.

Characteristics	Low expression of MRS2	High expression of MRS2	*P* value
n	277	277	
Clinical stage, n (%)			0.349
Stage I	180 (32.5%)	163 (29.4%)	
Stage II	27 (4.9%)	25 (4.5%)	
Stage III	58 (10.5%)	72 (13%)	
Stage IV	12 (2.2%)	17 (3.1%)	
Missing	0	0	
Primary therapy outcome, n (%)			0.922
PD&SD&PR	20 (4.1%)	18 (3.7%)	
CR	230 (47.7%)	214 (44.4%)	
Missing	27	45	
Age, n (%)			0.267
<= 60	110 (20%)	97 (17.6%)	
> 60	166 (30.1%)	178 (32.3%)	
Missing	1	2	
BMI, n (%)			0.153
<= 30	99 (19%)	113 (21.7%)	
> 30	164 (31.5%)	145 (27.8%)	
Missing	14	19	
Histological type, n (%)			**0.002**
Endometrioid	224 (40.4%)	188 (33.9%)	
Mixed	8 (1.4%)	16 (2.9%)	
Serous	45 (8.1%)	73 (13.2%)	
Missing	0	0	
Histologic grade, n (%)			**< 0.001**
G1	68 (12.5%)	31 (5.7%)	
G2	75 (13.8%)	46 (8.5%)	
G3	129 (23.8%)	194 (35.7%)	
Missing	5	6	
Tumor invasion(%), n (%)			0.828
< 50	141 (29.6%)	120 (25.2%)	
>= 50	114 (23.9%)	101 (21.2%)	
Missing	22	56	
Menopause status, n (%)			0.786
Pre	18 (3.6%)	17 (3.4%)	
Peri	10 (2%)	7 (1.4%)	
Post	229 (45.2%)	226 (44.6%)	
Missing	20	27	
Diabetes, n (%)			0.333
No	168 (37.1%)	161 (35.5%)	
Yes	57 (12.6%)	67 (14.8%)	
Missing	52	49	

**Notes.**

High and low expression were categorized according to the median expression value as a threshold, with patients in the high-expression group having MRS2 levels above the median and those in the low-expression group having MRS2 levels below the median. The bold styling indicates that these values are statistically significant.

### Identification of MRS2—associated mitochondrial genes in endometrial cancer

**Figure 5:**
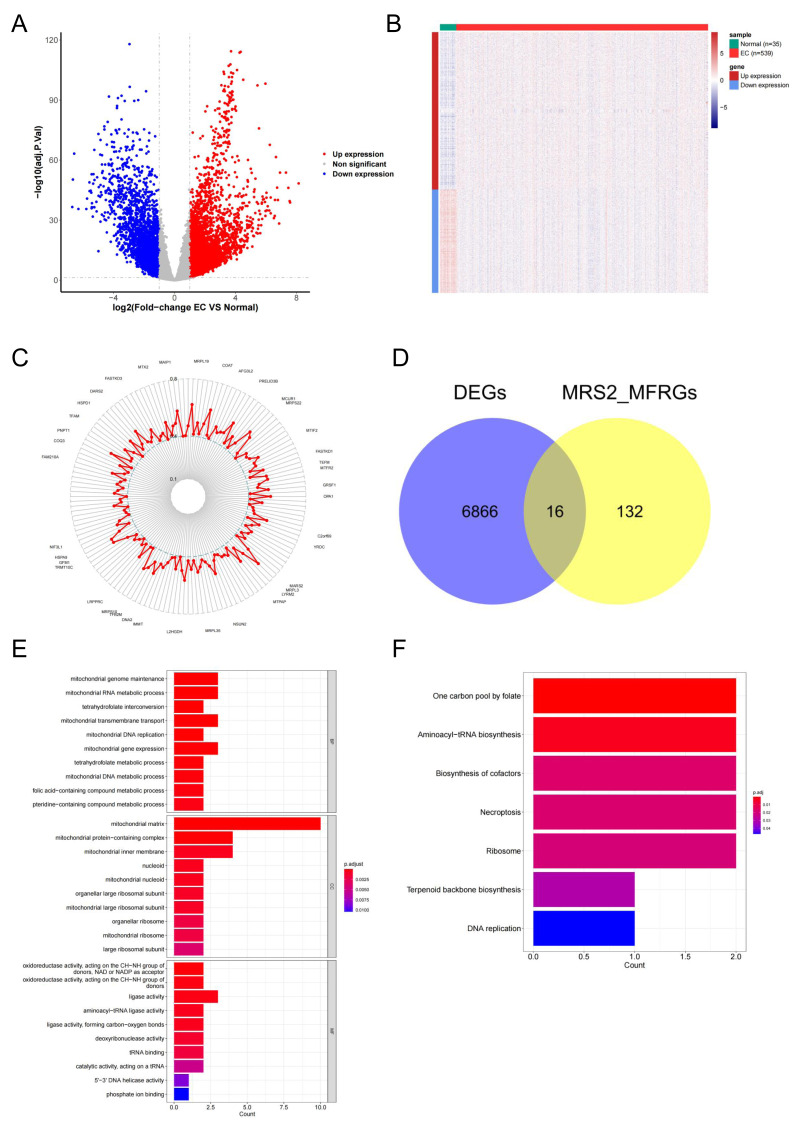
Identification of MRS2—associated mitochondrial genes in endometrial cancer. (A) Volcano map of differential genes. Red represents upregulation, blue represents downregulation. (B) Heat map of clustering of differential genes. (C) Mitochondrial function-related genes associated with the MRS2 gene. Each point represents a gene, and the position of the point represents the size of the correlation, smaller in the inside, larger in the outside. (D) The intersection of 6,880 differentially expressed genes (DEGs) and 148 MRS2-related mitochondrial function-related genes (MRS2_MFRGs). (E) Gene Ontology (GO) enrichment analysis. The horizontal axis is the number of genes enriched to each function, the left vertical axis is the GO functional items, and the right vertical axis is the functional classification. The color from red to blue indicates the significance of functional enrichment from high to low. (F) Kyoto Encyclopedia of Genes and Genomes (KEGG) enrichment analysis. The horizontal axis shows the number of genes enriched to each pathway, and the vertical axis shows the name of the KEGG pathway. The color from red to blue indicates the significance of functional enrichment from high to low.

To explore the functional relevance of MRS2 in endometrial cancer, we identified differentially expressed genes (DEGs) associated with mitochondrial function. Differential expression analysis using DESeq2 identified 6,880 DEGs in endometrial cancer (|logFC| ≥ 1, adj.*p* < 0.05), including 4,161 upregulated and 2,719 downregulated genes (Fig. 5A). The differences in gene expression are visualized in the heatmap shown in Fig. 5B.

By analyzing the association of the *MRS2* gene with genes related to mitochondrial function, 148 related genes were finally identified. The correlation results (Fig. 5C) exclusively demonstrated positive correlations, with the highest correlation coefficient being 0.6564 (*MRPL3*) and the lowest being 0.4008 (*AFG1L*).

The intersection of 6,880 DEGs and 148 MRS2-related mitochondrial function-related genes (MRS2_MFRGs) was obtained, as illustrated in Fig. 5D, yielding genes, including *DARS2, DNA2, FAM136A, MGME1, MRPL13, MRPL15, MTFR2, MTHFD1L, MTHFD2, PDK1, PDSS1, PGAM5, SLC25A13, SLC25A33, VDAC1,* and *YARS2*.

To comprehend the relevant biological functions and pathways of the candidate genes, visualization was conducted using the ggplot2 package, as demonstrated in Figs. 5E and 5F. GO analysis revealed that a total of 45 terms were enriched, including 16 cellular components (CC), seven molecular functions (MF), and 22 biological processes (BP). KEGG pathway analysis revealed that a total of seven functional pathways were significantly enriched, with most of these pathways related to mitochondrial function.

### Identification of MRS2—associated mitochondrial genes most closely linked to prognosis

**Figure 6:**
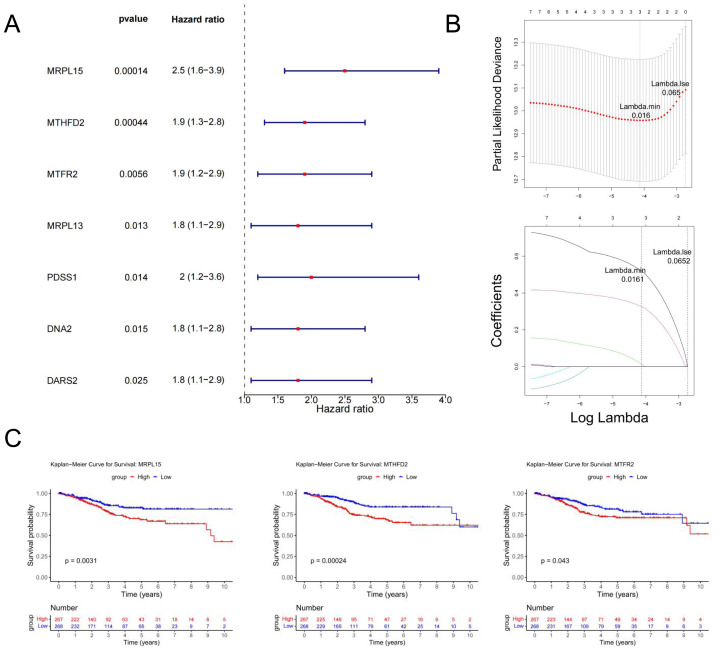
Identification of MRS2—associated mitochondrial genes most closely linked to prognosis. (A) Single-factor Cox regression analysis. In the left column, the names of genes significantly related to prognosis are listed in the first column, the second column is the *p* value, and the third column is the HR value (confidence interval). In the right column, the points in the figure are the HR value, green for negative and red for positive, and the horizontal line range is the confidence interval. (B) LASSO regression analysis. The figure above shows the curve of the penalty parameter, where x is the logarithm of lambda and y is the degree of freedom, which represents the cross-validation error; The horizontal axis below is log (*λ*), and the vertical axis is the coefficient of the gene, which shows the change of the coefficient of different variables after *λ* penalty. (C) Survival analysis. The continuous step curve, drawn with the horizontal axis as survival time and the vertical axis as survival rate, is used to illustrate the relationship between survival time and survival rate. The number in the lower coordinate axis (Number) of the figure is the number of samples surviving in the corresponding survival period.

Univariate Cox regression analysis identified seven genes (*MRPL15, MTHFD2, MTFR2, MRPL13, PDSS1, DNA2,* and *DARS2*) significantly associated with prognosis (*p* < 0.05) (Fig. 6A). These genes were further analyzed to identify key prognostic markers.

The glmnet package in R was employed to conduct Least Absolute Shrinkage and Selection Operator (LASSO) regression analysis on the seven genes identified above. The analysis selected the model with the minimum cross-validation error (lambda.min = 0.016), identifying three candidate key genes (*MRPL15*, *MTHFD2*, and *MTFR2*) (Fig. 6B).

Using the survival package in R, based on the expression levels of candidate key genes, EC samples were stratified into high and low expression groups (distinguished by the median), and survival analysis was conducted. The results (Fig. 6C) revealed significant differences in survival rates over time between the high and low expression groups for all three genes (*p* < 0.05). Therefore, *MRPL15*, *MTHFD2*, and *MTFR2* were identified as the key genes in this study.

### GSEA of key genes

**Figure 7:**
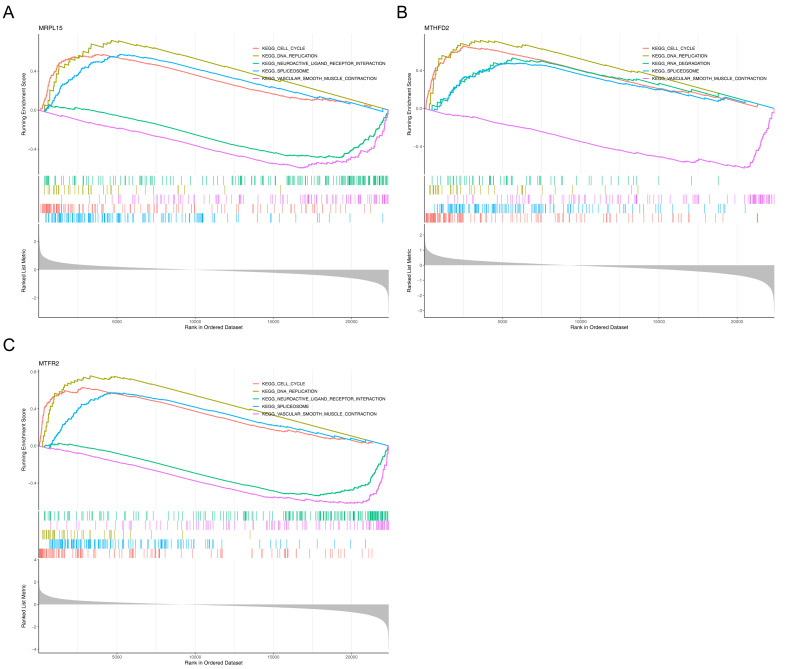
GSEA of key genes. (A) MRPL15 (B) MTHFD2 (C) MTFR2. GSEA enrichment trend chart. The top section illustrates the calculation process of enrichment scores (ES, enrichment score). From left to right, each gene generates an ES value that connects as lines. The most prominent peak at the far left represents the ES values for gene sets in phenotypic profiles. In the middle section, each line corresponds to a gene within the gene set and its position in the gene list. The bottom section displays a matrix illustrating the association between genes and phenotypes.

Gene Set Enrichment Analysis (GSEA) showed that the three genes were significantly enriched in cell cycle, DNA replication, spliceosome and vascular smooth muscle contraction pathways (Fig. 7), suggesting that they may be involved in related biological processes and play a key regulatory role in the development of endometrial cancer.

### Association of MRS2—related genes with immune infiltration and scoring in tumors

**Figure 8:**
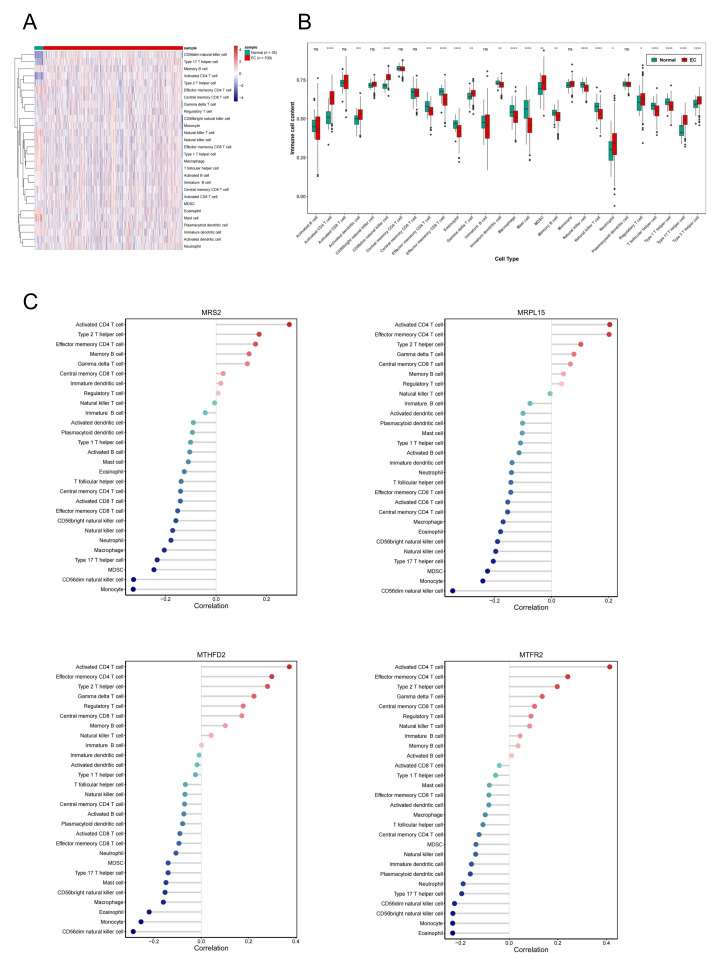
Association of MRS2-related genes with immune infiltration and scoring in tumors. (A) Heatmap of immune cell enrichment scores. The horizontal axis is the sample, and the vertical axis is 28 kinds of immune cells. The color represents the enriched fraction of immune cells after standardization, and the enriched fraction is from red to blue. (B) Wilcoxon tests for assessing the differences in immune cell infiltration levels between the normal and the EC group. The horizontal axis is immune cells and the vertical axis is enrichment fraction. Green represents normal group and red represents EC group. The top shows the significance of intergroup difference of immune cells. (C) Analysis of immune cell infiltration. (ns indicated *p* > 0.05, ^∗^ indicated *p* ≤ 0.05, ^∗∗^ indicated *p* ≤ 0.01, ^∗∗∗^ indicated *p* ≤ 0.001, ^∗∗∗∗^ indicated *p* ≤ 0.0001).

Single-sample gene set enrichment analysis revealed significant differences in immune cell infiltration between endometrial cancer and normal tissues (Fig. 8A). Among 28 immune cell types, 20 showed significant differences in enrichment scores (*p* < 0.05) (Fig. 8B), highlighting the role of immune microenvironment alterations in endometrial cancer progression. Next, we investigated the correlation between the expression levels of three key genes and MRS2 and the infiltration levels of 28 types of immune cells and visualized the results (Fig. 8C). The immune cells showing the highest positive correlation with these genes were activated CD4 T cells, effector memory CD4 T cells, and type 2 T helper cells.

**Figure 9:**
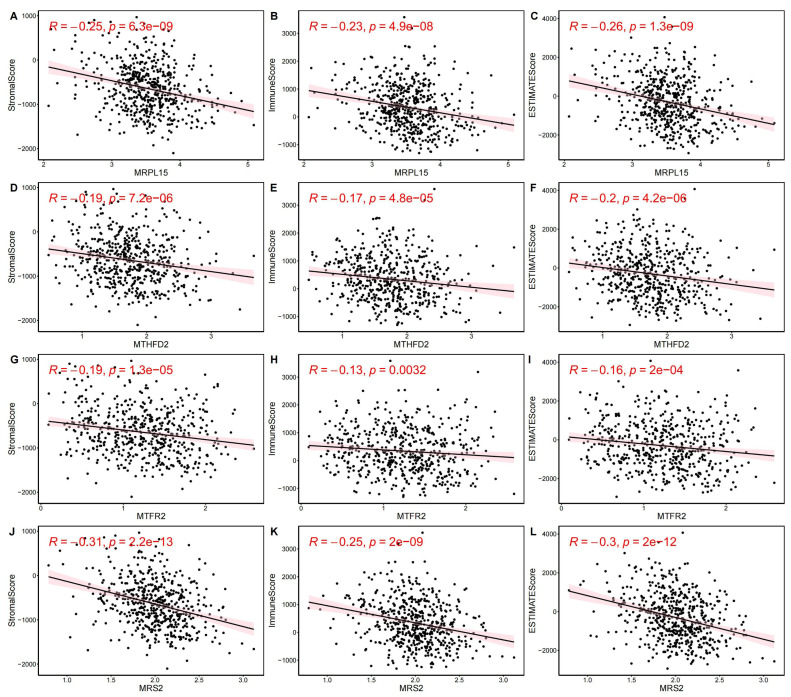
Correlation between key gene expression levels and tumor micro-environment scores. Correlation between key gene expression levels and tumor micro-environment scores. (A–C) MRPL15. (D–F) MTHFD2. (G–I) MTFR2. (J–L) MRS2.

Next, we evaluated EC samples in the training set using immune scores and stromal scores calculated based on the ESTIMATE algorithm. A total of three scores were predicted, namely Stromal Score (stromal score), Immune Score (immune score), and ESTIMATE Score (comprehensive score, the sum of the former two). We observed varying degrees of negative correlation between the four key genes and these three scores (*p* < 0.05). Among them, *MRPL15* exhibited the strongest negative correlation with all three scores, while *MTFR2* showed the weakest negative correlation (Fig. 9).

### Transcription factors of MRS2—associated mitochondrial genes

**Figure 10:**
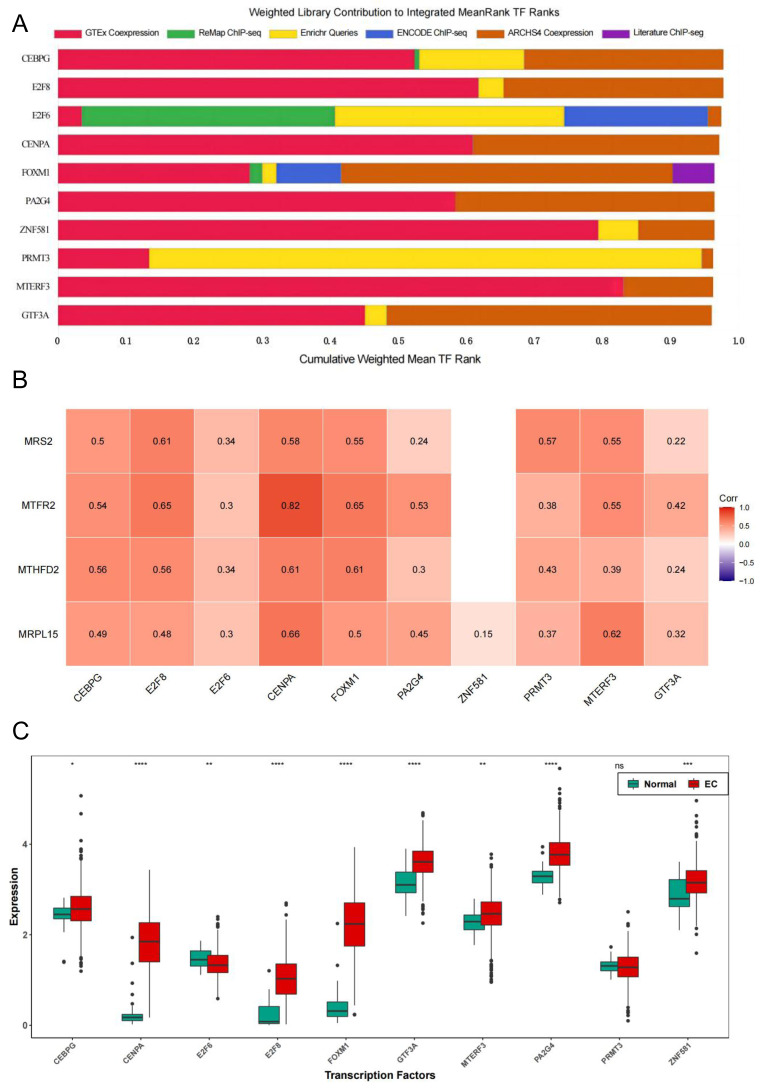
Transcription factors of MRS2—associated mitochondrial genes. (A) Mean rank composition of the top 10 transcription factors. The horizontal axis is the percentage and the vertical axis is the transcription factor. Different colors represent different sources of databases. (B) Correlation between key genes and transcription factors. The horizontal axis is the transcription factor and the vertical axis is the key gene. (C) Expression differences of transcription factors between the normal group and the EC group (ns indicated *p* > 0.05, ^∗^ indicated *p* ≤ 0.05, ^∗∗^ indicated *p* ≤ 0.01, ^∗∗∗^ indicated *p* ≤ 0.001, ^∗∗∗∗^ indicated *p* ≤ 0.0001).

To further explore the regulatory pathways of MRS2-related genes in endometrial cancer, we employed the ChIP-X Enrichment Analysis 3 transcription factor enrichment analysis tool (ChEA3) to predict the transcription factors associated with the three key genes. The composition of the top 10 transcription factors (CEBPG, E2F8, E2F6, CENPA, FOXM1, PA2G4, ZNF581, PRMT3, MTERF3, GTF3A) based on mean rank is illustrated in Fig. 10A. The correlation between the top 10 transcription factors and the four key genes is depicted in Fig. 10B, revealing varying degrees of positive correlation between the 10 transcription factors and the key genes. Except for the non-significant correlation between *ZNF581* and *MTFR2* and *MTHFD2*, all other relationships between the transcription factors and key genes were statistically significant (*p* < 0.05). The strongest correlation was observed between *CENPA* and *MTFR2* (0.8235).

Next, we analyzed the differential expression of the top 10 transcription factors between the Normal and EC groups using the Wilcoxon test. The results (Fig. 10C) revealed significant differences in the expression of all transcription factors between the groups, except for PRMT3, which did not exhibit significant differences in expression between the two groups (*p* < 0.05).

### Tumor drug sensitivity

**Figure 11:**
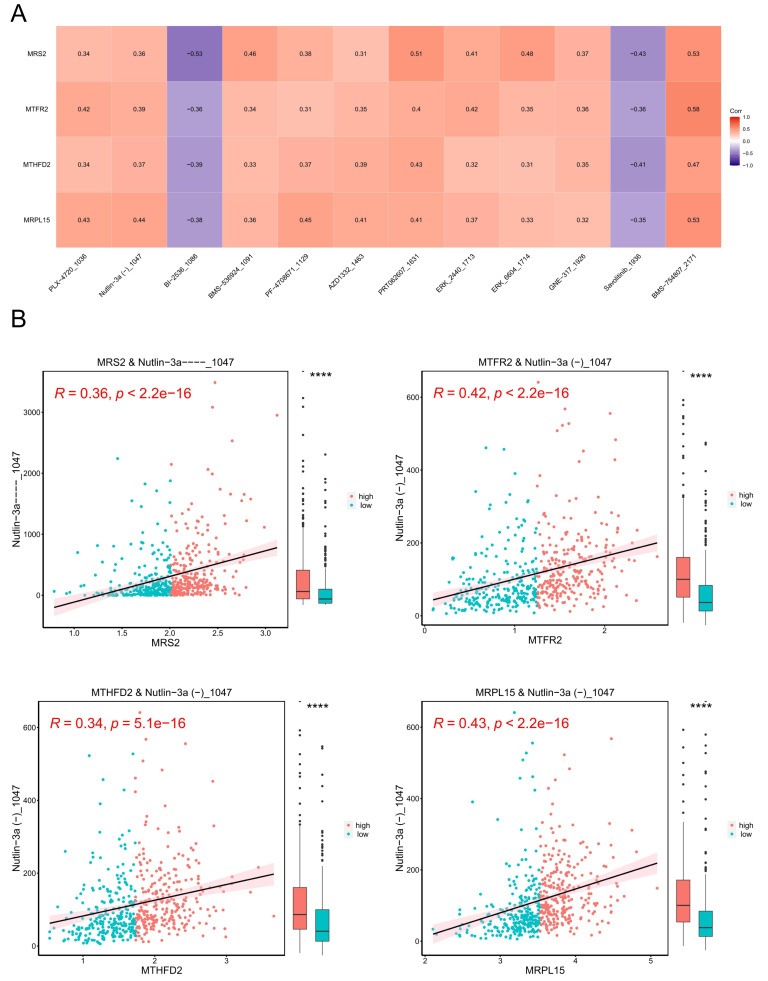
Tumor drug sensitivity. (A) Correlation between key genes and drug IC_50_. The horizontal axis is the drug and the vertical axis is the key gene. (B) Correlation between key genes and drug IC_50_. The horizontal axis represents genes, and the vertical axis represents drugs. Each point represents a sample, with red indicating the high-expression group and blue indicating the low-expression group. On the right is a box plot showing the distribution of drug IC_50_ in the high and low expression groups (^∗∗∗∗^ indicated *p* ≤ 0.0001).

A model was constructed using data from the Genomics of Drug Sensitivity in Cancer (GDSC) database (version 2), which includes data on 805 cell lines, 17,419 genes, and 198 drugs as the training set. The correlation between each drug’s IC_50_ values and key genes was calculated, and based on the criteria of —r— > 0.3 and *p* < 0.05, 13 drugs with higher correlations with key genes were selected. The results are presented in Fig. 11A.

Subsequently, we stratified EC samples into high and low expression groups based on the median expression levels of key genes. We observed differences in drug IC_50_ values between the high and low expression groups and generated a scatter plot depicting the relationship between key genes and drug IC_50_. The results (Fig. 11B) indicated that the drug BMS_754807_2171 exhibited the strongest positive correlation with *MRS2*, *MTFR2*, *MTHFD2*, and *MRPL15*.

### Protein expression validation

**Figure 12:**
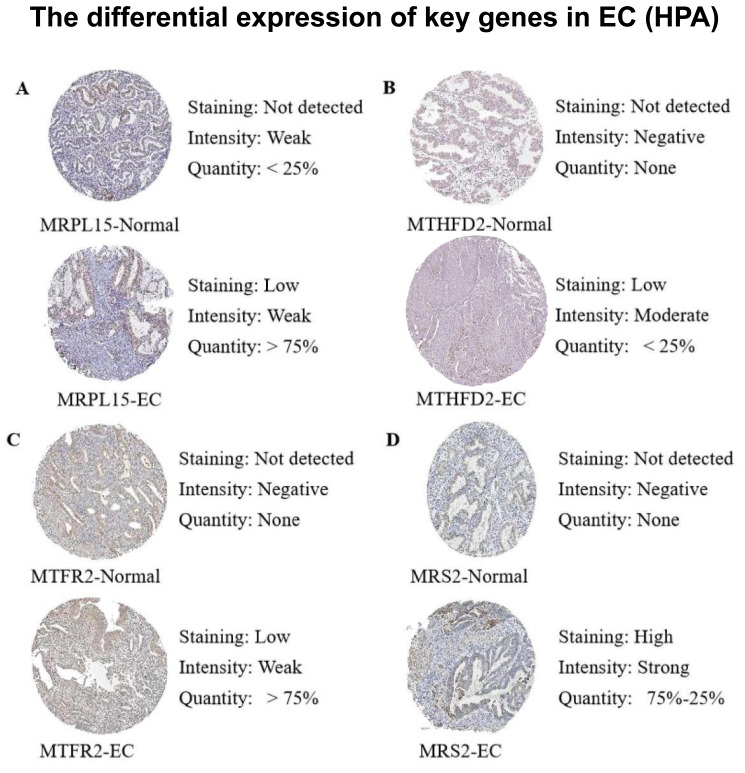
Differences in protein expression levels of key genes from the Human Protein Atlas (HPA) database. (A) The expression level of MRPL15 in EC and normal tissues. (B) The expression level of MTHFD2 in EC and normal tissues. (C) The expression level of MTFR2 in EC and normal tissues. (D) The expression level of MRS2 in EC and normal tissues.

Subsequently, we queried the expression profiles of these four pivotal genes at the protein level in both Normal and EC samples within the Human Protein Atlas (HPA) database, comparing their disparities. The results (Fig. 12) revealed that the protein expression levels were higher in EC samples compared with normal samples, consistent with the gene expression patterns elucidated in our previous analysis.

## Discussion

Mg^2+^ has been shown to be closely related to the occurrence and progression of many tumors (Lotscher et al., 2022), and a deficiency in magnesium can lead to mitochondrial oxidative stress and DNA damage (Huang et al., 2024). As a crucial mitochondrial magnesium ion transporter, MRS2 plays an indispensable role in regulating mitochondrial function and cellular metabolism (Daw et al., 2020).

This study demonstrated that MRS2 is overexpressed in multiple cancers, especially in endometrial cancer, where it correlates with poor prognosis and shows diagnostic potential (Mao et al., 2022). Recent work by Kolisek et al. (2003) revealed that MRS2 mediates magnesium transport from the endoplasmic reticulum to mitochondria under lactate stimulation, positioning magnesium as a key regulator of mitochondrial energy metabolism and MRS2 as its critical regulator. Additionally, three genes—MRPL15, MTFR2, and MTHFD2—were found functionally linked to MRS2, and their upregulation is associated with unfavorable outcomes in endometrial cancer patients. Together, MRS2 and related genes likely facilitate cancer progression by modulating mitochondrial magnesium levels and metabolic function.

Given the role of MRS2 in mitochondrial regulation, we further explored its mechanisms and found that high MRS2 expression elevated ROS levels. As mitochondrial-derived ROS are closely implicated in tumor progression—acting as a double-edged sword by promoting mutation, inflammation, and angiogenesis at high levels, while supporting redox signaling at lower levels—we hypothesized that MRS2 may influence tumor growth through ROS modulation. For instance, scavenging mitochondrial ROS has been shown to suppress tumor growth in Merkel cell carcinoma (Martins et al., 2024). Nevertheless, it is important to note that ROS may also exert tumor-suppressive effects, particularly through mechanisms such as Gasdermin-mediated pyroptosis (Shen et al., 2025). These findings collectively underscore the double-edged sword nature of ROS in tumor biology, capable of both promoting and suppressing tumor development in a context-dependent manner.

Wet-lab experiments confirmed that LPS-stimulated KLE cells exhibited increased lactate and corresponding upregulation of MRS2, consistent with prior reports (Daw et al., 2020). This was accompanied by a significant rise in ROS, supporting the role of MRS2 in mitochondrial ROS regulation and tumor progression in endometrial cancer. However, a paradoxical increase in ROS levels was observed alongside inhibited proliferation upon MRS2 knockdown, suggesting a complex regulatory role of MRS2 in redox homeostasis. These results indicate that MRS2 is essential for LPS-induced proliferation, and furthermore reveal a dichotomous role of ROS in this process. This observation aligns with the previously discussed double-edged sword nature of ROS, further supporting the context-dependent role of ROS in cancer cell regulation.

Subsequently, we identified the transcription factors of four mitochondrial functional genes. Although comprehensive mechanistic studies on MRS2 and its related genes remain to be conducted, the transcription factors provide new targets and a research foundation for future endometrial cancer diagnosis.

Given the emerging importance of immunotherapy in endometrial cancer (Mahdi, Chelariu-Raicu & Slomovitz, 2023), we also explored the relationship between MRS2 and its associated genes with tumor immunity. Notably, activated CD4+ T cells showed a positive correlation with all four genes, including MRS2, while NK cells and macrophages were negatively correlated. High infiltration of CD4+ T cells may promote immune evasion through PD-1/PD-L1 interactions (Herzog et al., 2015), suggesting that patients with elevated MRS2 pathway activity might benefit from immunotherapy.

Additionally, reduced NK cell levels were observed in patients with high MRS2 and related gene expression, consistent with impaired immune surveillance and poorer prognosis (Su et al., 2022a). While NK cell-based therapies are still developing, promising approaches—such as cord blood-derived NK cells (Mu et al., 2023)—highlight their potential. Although based on limited clinical evidence, these findings underscore the value of MRS2 and related genes as biomarkers for identifying patient subgroups likely to respond to immunotherapy, supporting more precise and personalized treatment strategies.

Finally, we investigated the influence of MRS2 and related mitochondrial genes on drug sensitivity and identified the IGF1R inhibitor BMS-754807 as the compound most strongly correlated with their expression. This suggests that endometrial cancer patients with high MRS2 expression may exhibit increased sensitivity to this inhibitor. Although BMS-754807 has not been widely applied in endometrial cancer, it has demonstrated synergistic anti-tumor effects in other cancers, such as non-small cell lung cancer, and is clinically available. Our findings support its potential as a targeted therapeutic option for endometrial cancer patients exhibiting high MRS2 pathway activity (Liu et al., 2018; Li et al., 2023).

Overall, MRS2 and associated genes represent a potential diagnostic and prognostic biomarkers for endometrial cancer. Their use may improve patient stratification and support personalized treatment strategies. However, given the context-dependent roles of ROS in tumor progression, the functional mechanisms of MRS2 require further elucidation. The precise regulatory relationship between MRS2 and ROS, as well as the functional interplay among MRS2, ROS, and endometrial cancer progression, warrants further investigation and validation. The current findings are primarily derived from *in vitro* models, which lack the complexity of the tumor microenvironment and cannot fully recapitulate the dynamic intercellular interactions occurring *in vivo*. Future studies should employ *in vivo* models and patient-derived xenografts to validate these relationships, and clinical trials targeting MRS2-related pathways may offer novel therapeutic opportunities.

## Materials and Methods

### Cell culture

Human KLE endometrial carcinoma cells (KeyGEN, Jiangsu, China) were cultured in PRMI-1640 containing 1% penicillin–streptomycin (Gibco, Billings, MT, USA) and 10% fetal bovine serum (FBS) (Gibco, Billings, MT, USA) at 37 °C in a humidified atmosphere (5% CO_2_, 95% air).

### Quantification of lactate content

Lactate concentration was measured enzymatically using the VITROS^^®^^ 5600 (Johnson & Johnson, New Brunswick, NJ, USA). Blood samples were immediately placed on ice after collection and centrifuged at 4 °C within 15 min to separate plasma. The enzymatic, solid-phase colorimetric method employed the VITROS Chemistry Products LAC Slide. Briefly, a 10 µL sample aliquot was dispensed onto the slide, and the subsequent enzymatic reaction, involving lactate oxidase and peroxidase, generated a colorimetric change. The rate of this change, measured by reflectance spectrophotometry at 540 nm, was kinetically monitored and quantified against a stored calibration curve.

### Cell counting kit-8

The cell suspension was prepared and inoculated into a 96-well plate with six repetitive wells. After adhering to the wall, LPS (catalog numbers: MB5198, Meilunbio, Dalian, China) was added to each well and cultured in the incubator at 37 °C for a corresponding time. A total of 10 ul Cell counting kit-8 (CCK8) enhanced solution (Meilunbio, Dalian, China) was added to each well subsequently. After further incubation for 0.5–4 h, the absorbance was measured at 450 nm.

### Flow cytometry detection for ROS

Cells were seeded and cultured to a confluence of 50–70% at the time of detection. A 10 µM final concentration of DCFH-DA (Meilunbio, Dalian, China) was prepared by diluting it in serum-free culture medium at a ratio of 1:1,000. The cell culture medium was then removed and a suitable volume of the diluted DCFH-DA working solution was added. The cells were incubated in the dark at 37 °C for 20–30 min. Following incubation, the cells were washed three times with serum-free culture medium to remove any uninternalized DCFH-DA. After collection, the cells were analyzed using a BD Canto II flow cytometer (Becton, Dickinson and Company, America). The fluorescence intensity was measured in real-time or at specific time points using an excitation wavelength of 488 nm and an emission wavelength of 525 nm.

### Transwell migration assay

Cells were resuspended in serum-free medium at a concentration of 5 ×10^4^ cells/mL. A total of 600 µL of medium containing 20% fetal bovine serum was added to the bottom wells of a 24-well plate. A Transwell insert with an 8-µm pore membrane was placed in each well, and 200 µL of the cell suspension was added to the top chamber. The device was incubated at 37 °C with 5% CO_2_ for 12–24 h. Non-migrated cells were removed, and migrated cells were fixed with 4% paraformaldehyde for 10 min. Cells were then stained with 1% crystal violet for 25 min. After washing and drying, the migrated cells were counted under a microscope to assess migration. Stained cells were imaged at 200 × magnification using an inverted microscope (Olympus, Tokyo, Japan).

### Cell immunofluorescence

A 12-well plate was prepared with 5µl of culture medium, and cell coverslips were placed for subsequent fixation. Cells in the logarithmic growth phase were harvested, digested with trypsin, and then resuspended in medium for counting. Following this, the cells were inoculated into a 12-well plate. The supernatant was discarded, and the cells were washed three times with PBS. Fixation was performed by adding 4% paraformaldehyde for 15 min at room temperature, followed by three washes with PBS, each lasting 5 min. The cells were then permeabilized with 0.5% Triton X-100 for 20 min at room temperature and washed again three times with PBS for 5 min each. To block nonspecific binding, an appropriate amount of 10% goat serum blocking solution (diluted with PBS) was added, and the cells were incubated for 2 h at room temperature. After the blocking solution was removed, the primary antibody, MRS2 (catalog numbers: DF14799, 1:200, Affinity Biosciences, Cincinnati, OH, USA), was added, and the cells were incubated overnight at 4 °C. Following three washes with PBS, the secondary antibody (catalog numbers: YM2006, AbFluor 488, 1:1,000, Immunoway, San Jose, CA, USA) was added. The cells were incubated in the dark for 60 min at room temperature before the secondary antibody was removed and the cells were washed three times with PBS for 5 min each. Incubation with DAPI was performed in the dark for 5 min, followed by the removal of the DAPI and three final washes with PBS, each lasting 5 min. The coverslip was then removed, blotted dry with filter paper, and inverted onto a slide containing anti-fade reagent. The coverslip was mounted using a sealing agent. Finally, images were captured using a fluorescence microscope (Nikon, Tokyo, Japan) at a wavelength of 555 nm.

### Transmission electron microscope

For transmission electron microscope (TEM) analysis, cultured cells were first fixed with 2.5% glutaraldehyde at room temperature for 5 min. Following fixation, cells were gently scraped in a unidirectional manner using a cell scraper to prevent membrane damage. The resulting cell suspension was transferred to a centrifuge tube and pelleted by low-speed centrifugation (≤3,000 rpm) for 2 min to obtain a compact cell aggregate approximately 3–4 mm in diameter. After replacing the initial fixative with fresh electron microscopy-grade fixative, the cell pellet was carefully resuspended and maintained in solution. Ultrastructural examination was subsequently performed using a HITACHI HT7700 transmission electron microscope (HITACHI, Tokyo, Japan).

### Data source

Training Set: RNA-seq data (count and FPKM values) and clinical information for EC and normal samples were downloaded from the TCGA-UCEC dataset using the TCGAbiolinks package in R. After removing duplicates, 574 samples (539 EC and 35 normal) were retained for analysis. In addition, the FPKM data of the training set were tested for normality, and the obtained *p*-values were used to determine whether the data conformed to a normal distribution. When the *p*-value was greater than 0.05, the null hypothesis was rejected, indicating that the data conformed to a normal distribution.

Validation set: The GSE63678 dataset was downloaded from the Gene Expression Omnibus (GEO) database (https://www.ncbi.nlm.nih.gov/geo/query/acc.cgi?acc= GSE63678), consisting of 35 samples, including 12 samples of EC and their controls (EC = 7, Normal = 5). This dataset was utilized to validate the expression levels of key genes.

Mitochondrial Function-Related Genes: A total of 1,136 mitochondrial function-related genes (MFRGs) were obtained from the human MitoCarta3.0 database (https://www.broadinstitute.org). Besides, 1,967 mitochondrial function-related genes were acquired from the Integrated Mitochondrial Protein Index database (IMPI, https://www.mrc-mbu.cam.ac.uk/). After taking the intersection of the two sets, 1,070 mitochondrial function-related genes were identified.

### Differential expression analysis

Differential gene expression analysis was performed using the DESeq2 package (1.36.0) in R (Love, Huber & Anders, 2014), with significant genes identified based on |logFC| ≥ 1 and adj.*p* < 0.05. Gene expression patterns were visualized using heatmaps generated with the pheatmap package.

### Candidate gene identification

To explore the role of MRS2 and mitochondrial function-related genes in the pathogenesis of EC, using the psych package in R, the relationship between the *MRS2* gene and mitochondrial function-related genes was analyzed based on Pearson correlation. Mitochondrial function-related genes associated with the *MRS2* gene were selected using the criteria of |r| > 0.4 and *p* < 0.05.

### Functional enrichment analysis

Functional enrichment analysis of disease-associated differentially expressed genes, including gene ontology (GO) and Kyoto Encyclopedia of Genes and Genomes (KEGG) pathways, was performed using the clusterProfiler package (4.4.4) in R (Wu et al., 2021). Significantly enriched functional entries and pathways were identified with a significance threshold of *p* < 0.05.

### Survival analysis

To screen for key genes significantly associated with survival, univariate Cox regression analysis (survival package (3.4-0) (MGrambsch, 2000) in R, *p* < 0.05) identified prognosis-related genes. LASSO regression analyses were performed using the glmnet software package (4.1-4) (Friedman, Hastie & Tibshirani, 2010), and the parameter with the smallest error was determined by 10-fold cross-validation (lambda.min = 0.016), and the results were screened accordingly to further screen candidate genes. To evaluate the prognostic significance of the key genes, the survival (3.4-0) (MGrambsch, 2000) and survminer packages (0.5.0) in R were used to classify the candidate key genes into high and low expression groups according to the median expression value. Kaplan–Meier survival curves were plotted, and genes with a significant correlation with survival (*p* < 0.05) were identified as the final key genes.

### Gene set enrichment analysis

To understand the biological functions and signaling pathways associated with key genes, samples were divided into high and low expression groups based on the median expression level of marker genes, followed by differential expression analysis. GSEA was then conducted using the C2: KEGG gene set from the MSigDB database, obtained *via* the msigdbr R package, as the background reference, and enrichment analysis was conducted on the ranked differentially expressed genes in the background gene set using the GSEA function in R (adj.*p* < 0.05).

### Analysis of immune infiltration

Immune cell infiltration levels were analyzed using ssGSEA (GSVA package in R), with enrichment scores calculated for 28 immune cell types. Wilcoxon tests were used to compare infiltration levels between EC and normal samples (A *p*-value less than 0.05 indicated statistical significance). The ggdotchart function of the ggpubr package (0.4.0) was used to visualize the correlation between three key gene expression levels and 28 levels of immune cell infiltration in a lollipop plot.

### ESTIMATE score

The estimate package in R was used to estimate the following scores in the EC samples of the training set: StromalScore, ImmuneScore, and ESTIMATEScore, with the ESTIMATEScore being the sum of StromalScore and ImmuneScore. A *p*-value < 0.05 indicated that the difference in the level of immune infiltration between the groups was statistically significant.

### Transcription factor identification and drug sensitivity screening

Transcription factors associated with the key genes were predicted using the ChEA3 tool (https://maayanlab.cloud/chea3/), and the top 10 significantly enriched were screened according to a significance threshold of *p* < 0.05 to analyze the differences in distribution between groups and the correlation between transcription factors and expression levels of key genes. This study utilized the oncoPredict software package (0.2) (Maeser, Gruener & Huang, 2021) in R to predict the IC_50_ values of multiple drugs in EC samples. The research employed version 2 data from the Cancer Drug Sensitivity Genomics Database (GDSC) as the training dataset, which contains 805 cell lines, 17,419 genes, and 198 drugs. A correlation model was established to analyze the relationship between drug HIC values and key genes. Drugs showing significant correlations with key genes (as indicated by correlation coefficients |r| > 0.3 and *p* < 0.05) were identified through computational analysis.

### Protein expression validation

To validate our findings at the protein level, immunohistochemical staining data for the key genes in endometrial tissue were retrieved from the Human Protein Atlas (HPA) database (https://www.proteinatlas.org/). The protein expression patterns were compared with transcriptional data to evaluate their consistency.

### Statistical analysis

Statistical analyses were performed using IBM SPSS Statistics 20 (IBM Corp., Armonk, NY, USA) GraphPad Prism 8, and R 3.5.1. Data are presented as mean ± SEM. Statistical comparisons between three or more groups were performed using one-way ANOVA for normally distributed data or the Kruskal–Wallis test for non-normally distributed data. Pearson correlation was used for correlation analysis, with *p* < 0.05 considered statistically significant.

## Conclusion

In summary, our findings suggest a potential association between MRS2 and its related mitochondrial genes with endometrial cancer progression, possibly through their involvement in mitochondrial metabolic processes and tumor immune regulation. Statistical analyses further revealed that elevated expression levels were significantly associated with higher tumor grade and poorer prognosis in patients. Besides, drug sensitivity analyses revealed that MRS2 and its related genes represent potential therapeutic targets, offering new treatment options for endometrial cancer patients.

##  Supplemental Information

10.7717/peerj.20409/supp-1 Supplemental Information 1 Data for cell experiments February 2025

10.7717/peerj.20409/supp-2 Supplemental Information 2 Data for cell experiment September 2025

## Additional Information and Declarations

### Data Availability

The following information was supplied regarding data availability: The data is available in the Supplemental Files. The raw data is available at Zenodo: - Lin. (2025). Raw Data [Data set]. Zenodo. https://doi.org/10.5281/zenodo.17239189
- Huang. (2025). Datas of IF and Transwell [Data set]. Zenodo. https://doi.org/10.5281/zenodo.14977272
The data is available at the mitochondrial function-related genes (MFRG) Repository via the Broad Institute: https://personal.broadinstitute.org/scalvo/MitoCarta3.0/human.mitocarta3.0.html
The data is available at the Integrated Mitochondrial Protein Index database (IMPI) via the University of Cambridge: https://www.mrc-mbu.cam.ac.uk/research-resources-and-facilities/impi
The raw sequence data are available at NCBI BioProject: GSE63678
